# PI3K/AKT/mTOR pathway plays a major pathogenetic role in glycogen accumulation and tumor development in renal distal tubules of rats and men

**DOI:** 10.18632/oncotarget.3675

**Published:** 2015-04-16

**Authors:** Silvia Ribback, Antonio Cigliano, Nils Kroeger, Maria G. Pilo, Luigi Terracciano, Martin Burchardt, Peter Bannasch, Diego F. Calvisi, Frank Dombrowski

**Affiliations:** ^1^ Institut für Pathologie, Universitätsmedizin Greifswald, Germany; ^2^ Klinik für Urologie, Universitätsmedizin Greifswald, Germany; ^3^ Molekularpathologie, Institut für Pathologie, Universitätsspital Basel, Switzerland; ^4^ German Cancer Research Center, Heidelberg, Germany

**Keywords:** AKT/mTOR, nephrocarcinogenesis, glycogen, clear cell tubules

## Abstract

Activation of the PI3K/AKT/mTOR pathway is a crucial molecular event in human clear cell renal cell carcinoma (ccRCC), and is also upregulated in diabetic nephropathy. In diabetic rats metabolic changes affect the renal distal tubular epithelium and lead to glycogen-storing Armanni-Ebstein lesions (AEL), precursor lesions of RCC in the diabetes induced nephrocarcinogenesis model. These lesions resemble human sporadic clear cell tubules (CCT) and tumor cells of human ccRCC.

Human sporadic CCT were examined in a collection of 324 nephrectomy specimen, in terms of morphologic, metabolic and molecular alterations, and compared to preneoplastic CCT and RCC developed in the rat following streptozotocin-induced diabetes or N-Nitrosomorpholine administration. Diabetic and non-diabetic rats were subjected to the dual PI3K/mTOR inhibitor, NVP/BEZ235.

Human sporadic CCT could be detected in 17.3% of kidney specimens. Human and rat renal CCT display a strong induction of the PI3K/AKT/mTOR pathway and related metabolic alterations. Proteins involved in glycolysis and *de novo* lipogenesis were upregulated. In *in vivo* experiments, dual inhibition of PI3K and mTOR resulted in a reduction of proliferation of rat diabetes related CCT and increased autophagic activity.

The present data indicate that human sporadic CCT exhibit a pattern of morphologic and metabolic alterations similar to preneoplastic lesions in the rat model. Activation of the PI3K/AKT/mTOR pathway in glycogenotic tubuli is a remarkable molecular event and suggests a preneoplastic character of these lesions also in humans.

## INTRODUCTION

In human clear cell renal carcinomas (ccRCC), the phosphoinositide 3-kinase (PI3K)/protein kinase B (AKT)/mammalian target of rapamycin (mTOR) cascade is frequently mutated and activated. Related metabolic alterations such as glycolysis, *de novo* lipogenesis and the pentose phosphate pathway are elevated, whereas gluconeogenesis, the adenosine-monophosphate activated protein-kinase (AMPK) pathway and the Krebs-cycle are decreased [1, 2]. Thus, this nutrient-sensing pathway is considered a potential therapeutic target in ccRCC, as mTOR inhibitors improve outcome of patients with metastatic RCC [1].

The PI3K/AKT/mTOR pathway is also upregulated in human diabetic nephropathy [3, 4]. Notably in this context, long-term type 2 diabetes mellitus is assumed to hold an increased risk of RCC development [5, 6]. Hyperglycemia associated mTOR activation leads to glomerular changes like hypertrophy, basement membrane thickening and mesangial proliferation, processes that can be ameliorated by rapamycin, a specific inhibitor of mTOR [3, 4].

In diabetic rats, morphological and metabolic changes affect mainly the renal distal tubular epithelium and lead to the development of glycogen storing clear cell tubules (CCT) [7]. They closely resemble glycogenotic tubules of the human diabetic kidney, known as Armanni-Ebstein lesions (AEL) [8, 9], whose cytoplasm is also filled with glycogen [10]. In long-term experiments, rat diabetes related CCT progressed to basophilic and acidophilic tubules, finally evolving to adenomas and carcinomas of clear, acidophilic or basophilic cell type, and thus representing very early preneoplastic alterations in rat nephrocarcinogenesis [7]. Clear cell preneoplastic lesions were also described in other rat models of chemically-induced nephrocarcinogenesis, i.e. after N-Nitrosomorpholine (NNM) administration, progressing to clear- and acidophilic RCC [11, 12, 13].

In rat diabetes related CCT, altered glucose metabolism has been well characterized, including upregulated glycogen synthesis and glycolysis and down-regulated glycogenolysis and gluconeogenesis, leading to the accumulation of glycogen. At the molecular level, strong upregulation of the insulin receptor, rat sarcoma/v-raf-1 murine leukaemia viral oncogene/mitogen-activated protein kinase (Ras/Raf-1/MAPK) cascade, transforming growth factor alpha (TGF-α), and epidermal growth factor receptor (EGFR) signaling was detected [7].

In humans, preneoplastic lesions of the kidney are scarcely described morphologically. Van Poppel reported “intratubular epithelial dysplasia” as a common precursor of sporadic RCC [14]. Nevertheless, only multiple cystic clear cell lesions in relation to the von Hippel-Lindau (VHL) disease are accepted as premalignant conditions [14, 15, 16]. These cysts reveal crucial genetic alterations like VHL gen mutations or allel deletion and an overexpression of the Hypoxia-inducible factor (HIF) [17, 18], which is a well-known oncogenic pathway in RCC [19].

While the importance of CCT in nephrocarcinogenesis has been demonstrated in the rat, the relevance of sporadically occurring human CCT has not been investigated intensively so far. Human glycogenotic tubules have merely been reported in the collecting duct system adjacent to invasive ccRCC by Cao et al. [20].

The importance of the PI3K/AKT/mTOR cascade in the initiation process of nephrocarcinogenesis remains unknown. As mentioned above, preneoplastic CCT of the diabetic rat reveal an early upregulation of protooncogenic pathways that are partially driven by unconstrained activity of the insulin receptor [7]. Insulin-related molecular alterations have been described in other preneoplastic lesions, like hepatocellular clear cell foci of the rat and in humans [21, 22]. In particular, both rat and human lesions displayed a sustained activation of the PI3K/AKT/mTOR cascade and associated metabolic alterations, including upregulated glycolysis, increased glycogen storage and *de novo* lipogenesis.

In the present study, we investigated the possible involvement of sporadic glycogenotic CCT in human renal carcinogenesis. For this purpose, we examined the morphologic, metabolic, and molecular similarities of renal CCT in rats and humans, with a particular emphasis on the role of the PI3K/AKT/mTOR cascade in these lesions.

## RESULTS

### Morphologic and metabolic features of rat diabetes related CCT

**Figure 1:**
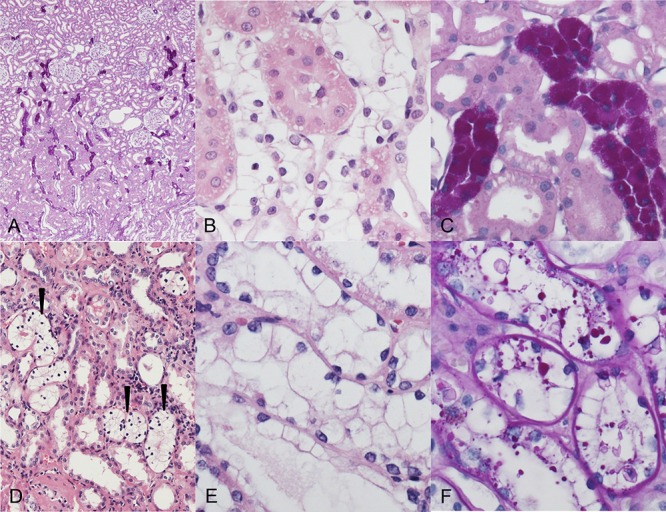
Clear cell tubules (CCT, A–C) of streptozotocin-induced diabetic rats and human glycogenotic alterations of distal tubules (D–F) **A.** Periodic acid-Schiff (PAS) reaction: distribution of PAS-positive rat AEL near the transition zone of renal cortex (upper part) and medulla (bottom). **B.** The cytoplasm of AEL cells is enlarged and appears clearly in hematoxylin and eosin (H & E) staining **C.** Magnification of PAS stain demonstrates the corresponding dense glycogen storage in distal AEL tubules. **D, E.** Human glycogenotic tubules (arrowheads) in a nephrectomy specimen. The similarity to rat CCT is easily recognizable in magnified H&E staining **F.** Glycogen is fast eluted in not perfused renal tissue, thus PAS staining is often patchy but still illustrates increased glycogen storage in enlarged tubules. Length of the lower edge A 1.25 mm, B, C, E, F 0.15 mm, D 0.5 mm.

**Figure 2:**
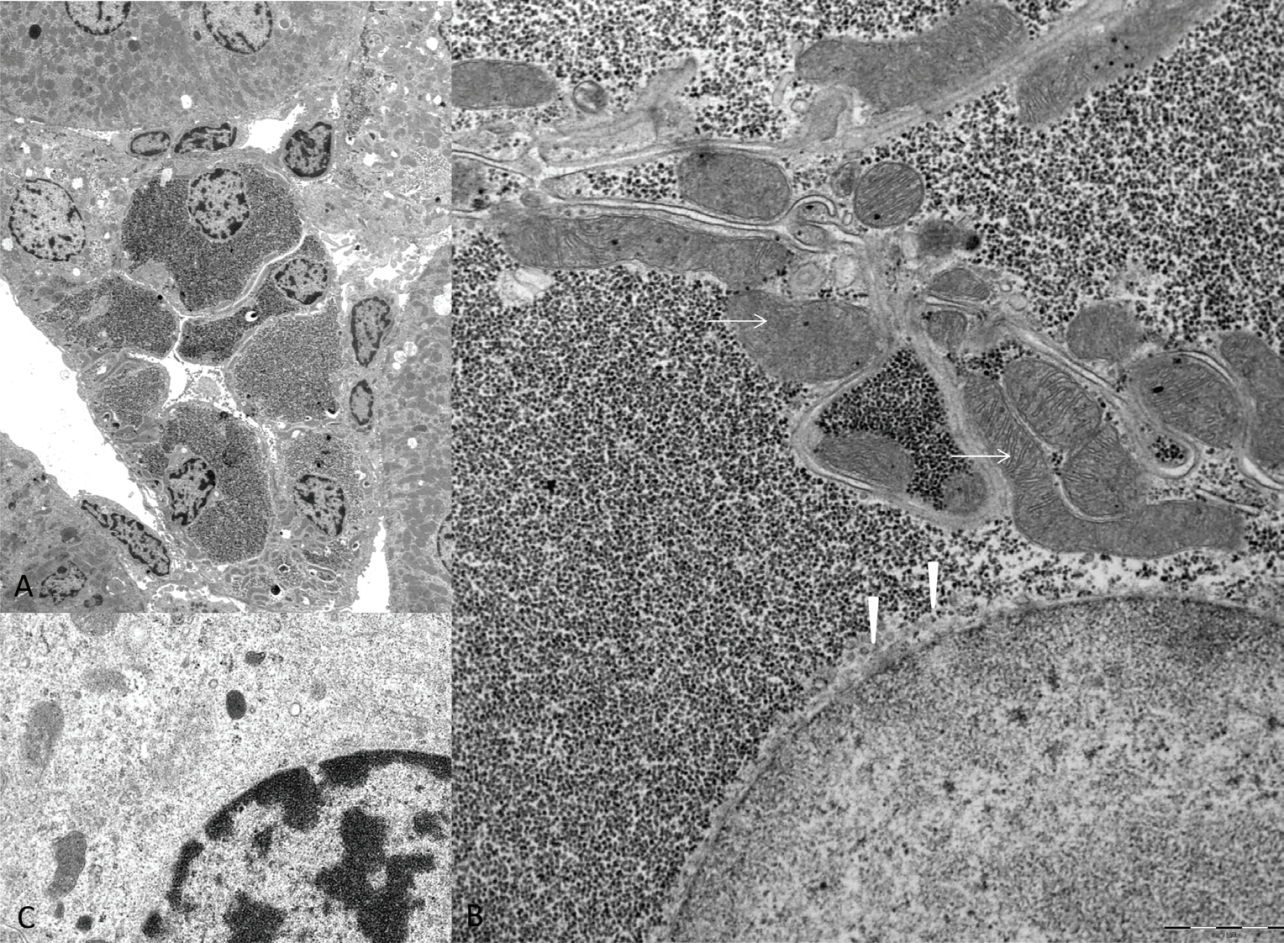
Electron microscopic features of diabetes related clear cell tubules (CCT) The overview micrograph **A.** illustrates a transverse section of an altered CCT with enlarged epithelium due to densely packed α-particles of glycogen in the cytoplasm **B.** in comparison to unaltered distal tubule epithelium **C.** with small amounts of glycogen and preserved loose distributed cell organelles. In CCT, masses of glycogen displace mitochondria (arrows) to the outer cell borders and the endoplasmatic reticulum (arrowheads) has nearly vanished and is only detectable beneath the nucleus. Length of the lower edge A 50 μm, B 7.5 μm, C 3 μm.

Rat diabetes related CCT consisted of groups of altered tubular epithelium with a clear cell character and could be detected in all diabetic rats, especially in the PAS-reaction as deep purple staining, accentuated at the junction of cortex and medulla (Figure 1). The morphology is due to the extensive glycogen storage, confirmed by the ultrastructural demonstration of dense α-glycogen particles in the cytoplasm (Figure 2). Acidophilic or basophilic changes did not occur at these early stages. Rat CCT showed a higher proliferative activity when compared to unaltered distal tubular epithelium of diabetic rats (BrdU-LI of AEL 0.99 ± 0.19% vs. DT 0.45 ± 0.16%, *p* < 0.05; Table 2).

**Figure 3:**
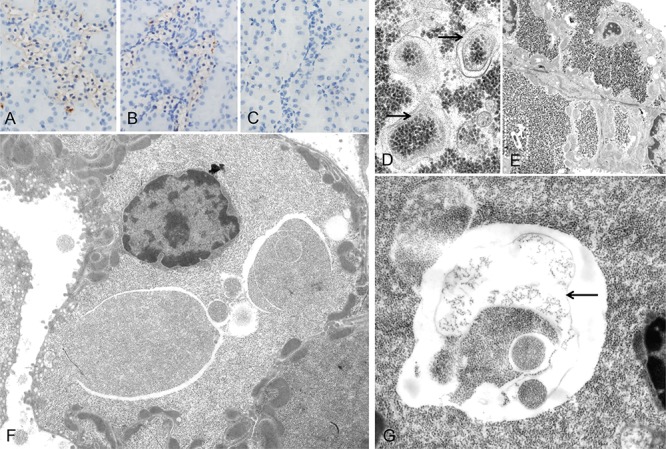
Autophagic activity in rat clear cell tubulus lesions Immunohistochemical demonstration of microtubule associated light chain 3 A (LC3A) indicates autophagy activity in glycogenotic tubules of diabetic rats, without **A.** or after treatment with NVP/BEZ235 for 4 weeks **B.** Low autophagy activity was detected in control kidney samples of non diabetic rats following NVP/BEZ235 administration for 4 weeks **C.** Electron microscopic examination reveals segregation vacuoles in the cytoplasm of the distal tubular epithelium with membrane bound vacuoles (**D.** arrows), containing glycogen particles, thus representing autophagic vacuoles. In untreated diabetic rats **E.** they are small and often singular (segmentated square). After treatment with NVP/BEZ235 for 4 weeks **F.**, **G.** vacuoles are multiple, large, filled with abundant amounts of glycogen and are membrane bound (arrow). Length of the lower edge: A-C 0.15 mm; D 1.5 μm, E 20 μm, F 17 μm, G 7 μm.

A basal autophagic activity within the cytoplasm of CCT of diabetic rats was detected, as substantiated by immunohistochemical demonstration of the autophagy marker protein, microtubule-associated light chain 3A (LC3A) (Figure 3). In addition, electron microscopy revealed the presence of membrane bound vacuoles containing large amounts of glycogen particles (Figure 3), thus representing autophagic vacuoles.

### Immunohistochemistry findings in rat diabetes related CCT

**Figure 4:**
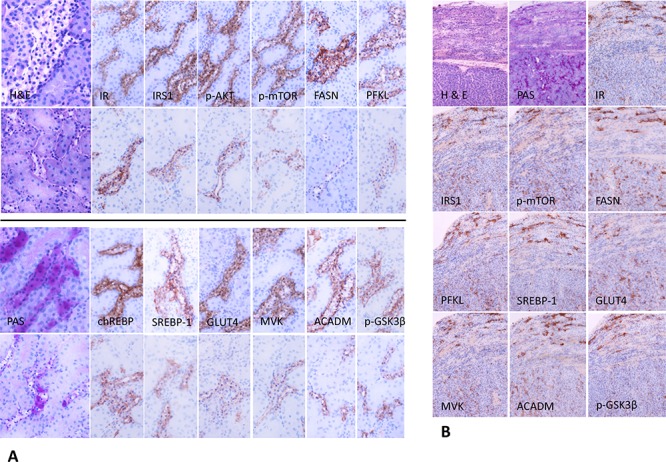
Representative immunohistochemistry in serial cryostat sections series of clear cell tubules (CCT) from streptozotocin-induced diabetic rats (A) and preneoplastic tubular lesions and renal carcinomas in rats after administration of N-Nitrosomorpholine (NNM) (B) **A.** CCT of diabetic rats after 4 weeks (upper panel of each row) with clear cell feature in H&E staining, which is caused by glycogen storage, as shown by strong positivity to the PAS reaction. Overexpression of the insulin receptor (IR) leads to upregulation of activated/phosphorylated AKT and mTOR proteins. Glucose accumulation is due to upregulation of glucose transporters, i.e. GLUT4, a process dependent on insulin. Utilization of glucose in the glycolytic pathway (PFKL) is facilitated by activated/phosphorylated AKT and chREBP. Glycogenolysis is inhibited, as GSK-3β is phosphorylated/inactivated. Additionally, SREBP-1 mediates transcription of enzymes of *de novo* lipogenesis (MVK, FASN). Simultaneously, β-oxidation of fatty acids is activated, as acyl-CoA dehydrogenase for acyl chains of medium length (ACADM) is upregulated. CCT of rats treated with NVP/BEZ235 for 1 week (lower panel of each row) are small and narrowed and reveal lower expression of all members of the PI3K/AKT/mTOR pathway examined. **B.** Molecular and metabolic alterations in NNM-induced glycogenotic preneoplastic tubules (upper part of each picture) and solid renal cell carcinomas (lower part) are similar to those detected in diabetes-induced nephrocarcinogenesis (24 months old rat, NNM was administered as a single dose). Length of the lower edge: 4A H&E, PAS 0.25 mm; immunohistochemistry 0.125 mm; 4B 0.3 mm.

CCT were compared via immunohistochemical reactions to preneoplastic tubulus changes and RCC induced by NNM administration. Noticeably, diabetes-induced CCT revealed the same immunohistochemical patterns that were detected in NNM-induced lesions (Figure 4). In particular, they showed a stronger cytoplasmic immunoreactivity for the insulin receptor (IR) when compared to the surrounding unaltered proximal and distal tubules. AEL overexpressed the glucose transporter proteins GLUT 1, 2 (not shown) and 4. In general, glycolysis mainly occurs in the distal part of Henle's loop [23]. CCT were characterized by an additional upregulation of the glycolytic enzymes phosphofructokinase (PFKL), pyruvate kinase 2 (PKM2, not shown) and glucokinase (IGLK, not shown), which are primarily activated by insulin-mediated signaling (Figure 4). Glycogenolysis was inhibited because glycogen-synthase-kinase 3β was inactivated by phosphorylation (*p*-GSK3β).

Since the PI3K/AKT/mTOR cascade is a pivotal effector of insulin signaling and a major inducer of glycolysis and lipogenesis and a repressor of glycogenolysis [24], this pathway was further investigated. Cells in CCT showed a strong immunoreactivity for phosphorylated/activated AKT and mTOR, phosphorylated/inactivated AMP activated protein kinase (AMPK), phosphorylated/activated ribosomal protein S6 (RPS6), and phosphorylated/inactivated 4E-BP1, when compared to normal kidney tissue. In contrast to normal distal tubules possessing low lipogenic activity [23], the main enzymes of *de novo* lipogenesis, including ATP-Citrate Lyase (ACLY), Acetyl-CoA Carboxylase (ACAC), fatty acid synthase (FASN), its stabilizing protein ubiquitin-specific protease 2A (USP-2A) (not shown), and stearoyl-CoA desaturase 1 (SCD1) were found to be upregulated in AEL (Figure 4). Similarly, levels of the carbohydrate responsive element binding protein (chREBP) and sterol regulatory element binding protein (SREBP-1)-transcription factors, which mediate the lipogenic effects of insulin, were elevated in AEL. Interestingly, the β-oxidation enzyme acyl-CoA dehydrogenase for acyl chains of medium length from 4–16 (ACADM) also showed a strong immunoreactivity in AEL.

### Morphologic, metabolic, and molecular features of human sporadic CCT

Next, we examined a collection of human renal specimens (*n* = 324) for the same parameters. Glycogenotic CCT could be detected in the renal cortex in 56 of the 324 kidney specimens (17.3%). They occurred as multiple lesions (defined as more than 10 CCT in one paraffin section of 1 cm^2^) in 22 of 56 specimens (39.3%). The frequency of CCT did not correlate significantly with the renal tumor type (clear cell, papillary, chromophobe RCC, or oncocytoma) or urothelial carcinoma (Table 1). On the other hand, in 69.9% of CCT containing kidneys the tumor type was a ccRCC. Of note, our collective did not include cases of VHL disease, and we did not find VHL typical clear cell cystic lesions.

**Table 1:** Frequency of clear cell tubules (CCT) in nephrectomy specimen and association to tumor entities

collective	*N*	CCT		multiple CCT*
	324	56/324 (17.3%) frequency	proportion of CCT	22/56 (39.3%) of multiple CCT
ccRCC	221	39/221 (17.2%)	39/56 (69.9%)	14/22 (63.6%)
papillary RCC	30	3/30 (10.0%)	3/56 (5.4%)	1/22 (4.5%)
chromophobe RCC	8	1/8 (12.5%)	1/56 (1.8%)	0
oncocytoma	17	6/17 (35.2%)	6/56 (10.7%)	4/22 (18.2%)
other RCC subtypes	4	0	0	0
urothelial carcinoma	22	5/22 (22.7%)	5/56 (8.9%)	3/22 (13.6%)
angiomyolipoma	2	0	0	0
neuroblastoma	2	0	0	0
other neoplasia (lymphoma, sarcoma)	4	1/4 (25.0%)	1/56 (1.8%)	0
non neoplastic kidney diseases	14	1(7.1%)	1/56 (1.8%)	0

* multiple CCT are defined as more than 10 CCT in one paraffin section of 1 cm^2^.

abbreviations: CCT, clear cell tubules; ccRCC, clear cell renal cell carcinoma.

To better define the origin of human CCT, some renal tubule antigens were investigated by immunohistochemistry. CCT exhibited immunoreactivity for cytokeratin 7 (CK7) at the periphery of their cytoplasm similar to the neighboring unaltered distal tubular epithelium, but revealed no apical CD10 expression like the proximal tubular system (Supporting Figure 4).

**Figure 5:**
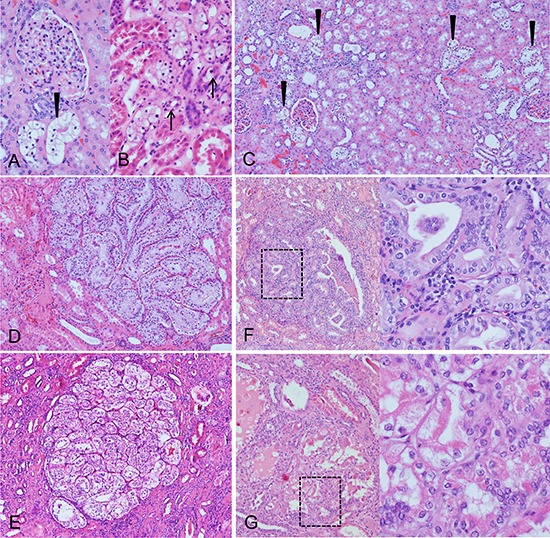
Morphological aspects of human clear cell tubules (CCT) and advanced lesions Glycogenotic CCT (**A.** arrowhead) at the related glomerulus with its vas afferens and the transition (**B.** arrows) of distal tubule epithelial cells to glycogen storing CCT and multiple CCT lesions in the renal cortex (**C.** arrowheads) could be detected in the nephrectomy specimens. The glycogenotic tubules also form cell clusters near the related glomerulus **D.** or almost 1 mm large nodules. Nodular lesions with a basophilic (**E.** larger magnification of the square box) or acidophilic (**F.** larger magnification of the square box) cytoplasm, enlargement of nuclei and prominent nucleoli represent advanced lesions. Length of the lower edge: A, B 0.3 mm, C 1.67, D, E 1 mm, F, G 0.6 mm.

CCT appear also as swollen tubulus epithelium with an “empty” looking cytoplasm in H&E staining, containing abundant glycogen, as demonstrated by the PAS reaction (Figure 1). Transition of distal tubule epithelial cells to glycogen storing CCT was evident. Furthermore, transformation of glycogenotic tubuli to small cell clusters or almost 1 mm large nodules occurred, thus representing advanced clear cell lesions. In rare cases, nodular lesions revealed a basophilic or acidophilic cytoplasm, enlargement of nuclei and prominent nucleoli, which can be interpreted as small adenomas (Figure 5).

**Figure 6:**
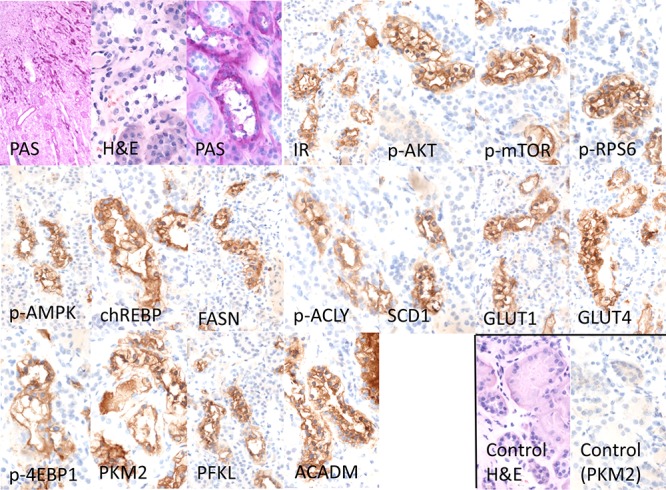
Representative immunohistochemical findings in serial cryostat sections series of human glycogenotic tubules At low magnification and PAS reaction, human glycogenotic distal tubules can be easily detected in the transition zone of renal cortex and medulla. Their cytoplasm is swollen and appears “empty”, containing glycogen, as it can be demonstrated with purple staining in PAS-reaction. The insulin receptor (IR) and members of the PI3K/AKT/mTOR signaling pathway (phosphorylated AKT and mTOR, RPS6, and 4EBP1) are induced when compared with adjacent renal tissue (control H&E and representative negative immunohistochemistry staining). Activation of the IR increases glucose uptake via translocation to the cell membrane of GLUT1 and 4. Also, glycolysis (PKM2, PFKL), *de novo* lipogenesis (phosphorylated/inactivated AMPK, FASN, ACLY, SCD1 and chREBP) and β-oxidation of lipids (ACADM) are strongly induced in glycogenotic tubules. There is no expression of any of the investigated proteins in proximal or unaltered distal tubules of the same kidney (control), as shown by representative immunohistochemistry for PKM2. Length of the lower edge: PAS overview 0.4 mm; remaining stainings 0.14 mm.

At the immunohistochemical level, the protein expression patterns of the PI3K/AKT/mTOR pathway (p-AKT, p-mTOR, p-RPS6, p-4EBP1) was equivalent in human CCT to those described for rat AEL (Figure 6). All proteins tested were upregulated in CCT when compared to unaltered tubules. The insulin receptor (IR) was found to be induced, and this was paralleled by increased levels of GLUT1 and 4. Furthermore, glycolysis (PKM2, PFKL) and *de novo* lipogenesis (p-AMPK, FASN, p-ACLY, SCD1, chREBP) were strongly induced in CCT when compared to unaltered tubules, as observed in rat CCT.

### Human ccRCC display the same metabolic and molecular alterations as those detected in rat and human CCT

Subsequently, representative members of the PI3K/AKT/mTOR pathway were assessed in TMAs of human ccRCC by immunohistochemistry (Supporting Table 4, Supporting Figure 2). PKM2, SCD1, ACAC, and ACLY proteins were upregulated regardless of tumor differentiation grade. Levels of FASN expression increased with tumor dedifferention (50% of G1 to 80% of G4 tumors). Levels of phosphorylated AKT and mTOR were strongly induced, mainly in lower differentiated ccRCCs (G3 and G4). Immunohistochemical findings were further substantiated by Western Blot analysis in a small collection of human ccRCC (Supporting Figure 3).

### The dual PI3K/mTOR inhibitor NVP/BEZ235 restrains proliferation of rat diabetes related CCT and human ccRCC cell lines

#### Effects *in vivo*

Treatment with NVP/BEZ235 reduced the proliferative activity of CCT in hyperglycemic rats by ~5 folds (0.19%) after 1 week and by ~3 folds (0.34%) after 4 weeks, respectively (Table 2). Furthermore, volume fraction of the autophagic vacuoles in rat glycogenotic tubules was increased. Volume fraction was 2 folds higher after 1 week (2.92%) and 3 folds higher after 4 weeks (5.01%) of NVP/BEZ235 treatment when compared to untreated rats (1.46%) (Table 3). Of note, treatment of diabetic rats with NVP/BEZ235 resulted in a distinct reduction of the staining intensity of the various proteins when compared to untreated diabetic rats (Figure 4)

**Table 2:** The effect of NVP/BEZ235 (NVP) on the proliferative activity of AEL in diabetic rats

	*N*	Proliferative activity (BrdU-LI) %		
		AEL	DT	PT
4 weeks diabetic, control	16	0.99 ± 0.19	0.45 ± 0.16*^1^	0.97 ± 0.16
4 weeks diabetic, 1 week NVP	11	0.19 ± 0.05*^3^	0.06 ± 0.03*^1^,^3^	0.95 ± 0.25*^2^
4 weeks diabetic 4 weeks NVP	11	0.34 ± 0.05*^3^	0.43 ± 0.13	1.31 ± 0.18*^2^

Rats were subjected to daily administration of the PI3K/mTOR dual inhibitor, NVP/BEZ235 at a concentration of 40 mg/kg for one week or 10 mg/kg body weight for 4 weeks. BrdU was administered as two injections 24 hours and 1 hour before sacrifice (s.c. and i.p.). AEL, Armanni-Ebstein lesions; DT, distal tubular epithelium; PT, proximal tubular epithelium; BrdU-LI: BrdU labeling index.

* *p* < 0.05:

1 DT vs. AEL

2 PT vs. AEL

3 NVP treatment vs. control

**Table 3:** Volume fraction of autophagic vacuoles and the effect of NVPBEZ235 (NVP)

AEL	*N*	Volume fraction of APV %
4 weeks diabetic	12	1.46 ± 0.24
4 weeks diabetic, 1 week NVP	9	2.92 ± 0.52*
4 weeks diabetic 4 weeks NVP	21	5.01 ± 0.45*

The volume fraction of large autophagic vacuoles (APV) in Armanni-Ebstein lesions (AEL) was estimated from their area fraction in semithin sections stained according to Richardson at x900 magnification. Rats were subjected to daily administration of the PI3K/mTOR dual inhibitor, NVP/BEZ235 at a concentration of 40 mg/kg for one or 10 mg/kg body weight for 4 weeks.

* *p* < 0.05, 1 and 4 weeks NVP vs. without NVP.

#### Effects *in vitro*

Treatment of human ccRCC cell lines CAKI1 and RCC4 with NVP/BEZ235 resulted in a strong decrease in cell proliferation and induction of apoptosis (Supporting Figure 1). At the molecular level, NVP/BEZ235 treatment suppressed phosphorylation of AKT protein and mTOR and of downstream effectors (S6RP and 4EBP1). Also, the key enzyme responsible for *de novo* lipogenesis, FASN, was downregulated following NVP/BEZ235 treatment.

## DISCUSSION

In this investigation, we demonstrate remarkable similarities between human sporadic CCT and rat clear cell precursor lesions in diabetes-induced renal carcinogenesis [7] and chemically induced nephrocarcinogenesis [11]. In particular, human CCT closely resemble the rat glycogenotic tubular lesions induced both by diabetes and NNM in terms of morphological, metabolic, and molecular alterations, suggesting a preneoplastic character of this tubulus morphology also in humans. We want to emphasize that we focused in this study on sporadic occurring CCT and not diabetes related AEL in humans.

**Figure 7:**
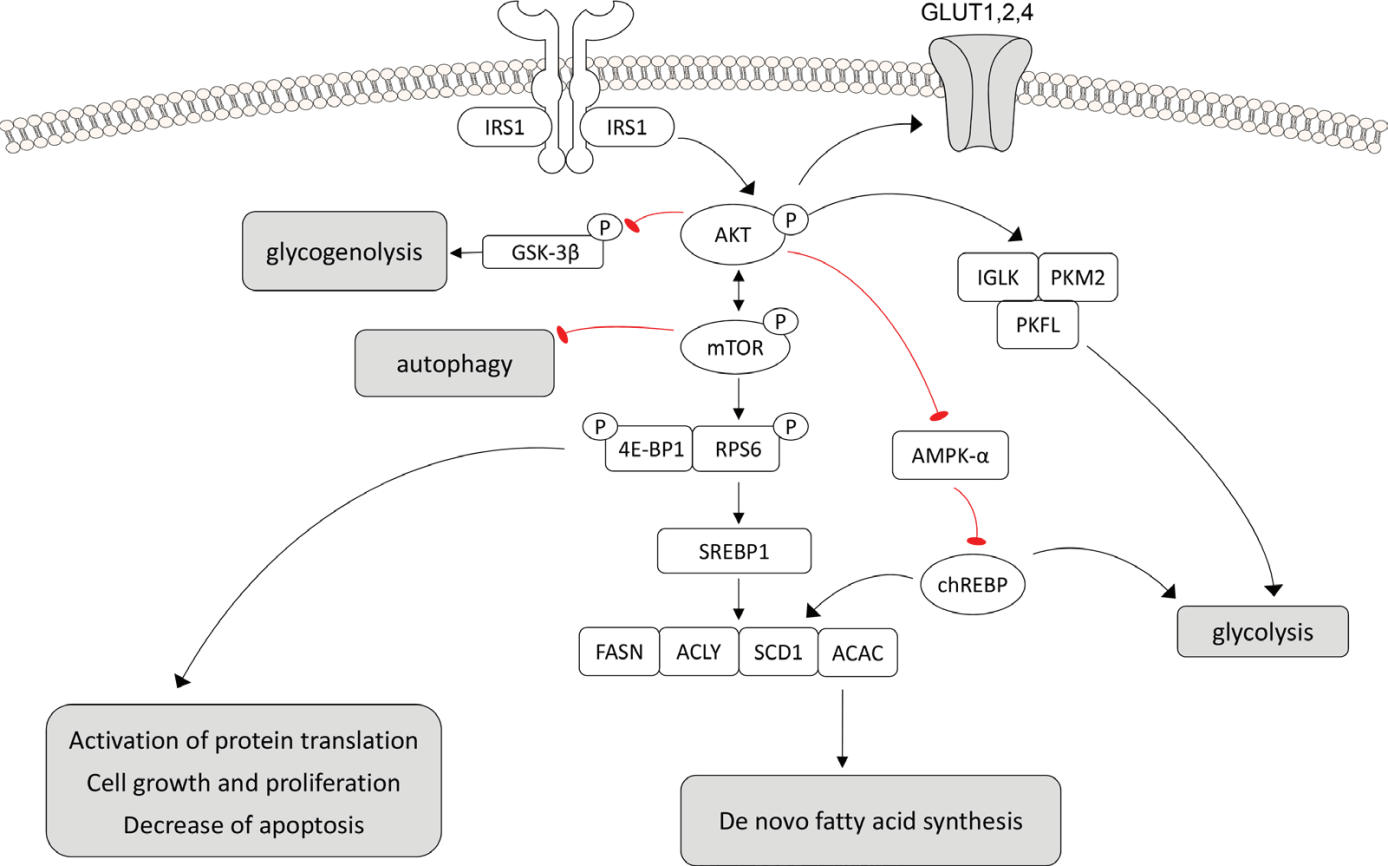
Schematic representation of the molecular mechanisms responsible for the glycogenotic and lipogenic phenotype and promotion of cell growth in rat preneoplastic clear cell tubulus (CCT) lesions, human clear cell renal cell carcinoma and presumably human CCT Deregulation of the PI3K/AKT/mTOR cascades induces *de-novo*-lipogenesis (chREBP, SREBP-1, p-ACLY, ACAC, FASN, USP-2A, SCD1), and glycolysis (IGLK, PKM2, PFKL). The glucose required for the aforementioned processes is obtained by the upregulation of GLUT1 and 4 glucose transporters. In addition, the AKT/mTOR cascade inactivates GSK-3β, AMPKα, 4E-BP1, and activates RPS6. Autophagy is also inhibited. Aberrant activation of lipogenesis and glycolysis by the PI3K/AKT/mTOR pathway leads to the upregulation of oncogenic signals, with consequent tubular epithelium proliferation and growth. Symbols: arrows: activation, blunted arrows: inhibition.

We show that the PI3K/AKT/mTOR cascade and related metabolic changes, including increased glycolysis, glycogen synthesis, and *de novo* lipogenesis, are induced in the very early stages of nephrocarcinogenesis in the rat and in human glycogenotic tubules. These peculiar metabolic alterations (Figure 7) resemble the Warburg effect in many cancer entities [25], and are also documented in human and rat preneoplastic glycogenotic foci of the liver [21, 22]. The same metabolic and molecular alterations occur in advanced stages of human ccRCC [1]. In particular, the PI3K/AKT/mTOR cascade is recurrently activated in ccRCC and strongly associated with ccRCC progression [26, 27]. Moreover, in diabetic nephropathy, there is an early activation of mTOR signaling leading to hypertrophy, proliferation and apoptosis in cells of the mesangium and proximal tubular cells [4].

We evaluated the relevance of the PI3K/AKT/mTOR signaling pathway in renal carcinogenesis by *in vitro* and *in vivo* approaches. Dual inhibition of PI3K/mTOR by NVP/BEZ235 led to a reduction of cell growth and increase of apoptosis in human CAKI1 and RCC4 cells, in accordance with previous findings [26, 27]. Furthermore, inhibition of this pathway resulted in a reduced proliferative activity of rat diabetes induced AEL. Taken together, the present data indicate that aberrant activation of the PI3K/AKT/mTOR cascade is a key alteration in early lesions along renal carcinogenesis.

Importantly, the finding of upregulated *de novo* lipogenesis but absent steatosis in altered rat and human glycogenotic tubules is novel. Of interest, in studies of diabetic nephropathy, fatty vacuolization in proximal tubulus regions [9] and accumulation of triglycerides [28] in the cortex and sometimes also in human AEL occur [29]. In our collection of human sporadic CCT, we did not detect any sign of lipid storage. Nevertheless, overexpression of lipogenic enzymes such as ACAC has been extensively described in ccRCC [1]. In addition, elevated expression of SCD1 [30, 31], which leads to the conversion of saturated fatty acids to monounsaturated fatty acids, is a predominant feature of ccRCC. As a consequence, SCD1 has been hypothesized as a potential therapeutic target for ccRCC [31]. Monounsaturated fatty acids are a major component of biological structures, such as cell membranes, and function as signaling molecules that are needed for cancer cells to proliferate and survive [31]. The lack of steatosis that we observed in CCT despite the upregulation of lipogenic proteins might be because the high amount of lipid derivates are promptly used by CCT cells for their metabolic needs. In accordance with the latter hypothesis, the important fatty acid oxidation protein, ACADM, is upregulated in rat and human CCT, while being downregulated in ccRCC.

Interestingly, the insulin receptor was found to be upregulated in human CCT in comparison to the surrounding tubule apparatus. Physiologically, the insulin receptor is expressed in glomerular podocytes and distal tubular regions, regulating sodium excretion and blood pressure control [32]. We detected the translocation of GLUT 4 to the cell membrane in CCT - a process that is regulated by insulin as well - thus providing a link between deregulation of insulin signaling and development of glycogenosis and lipogenic phenotype of tubular epithelium also in humans. However, diabetes-driven nephrocarcinogenesis could not be substantiated in humans to date and requires further investigations, as long-term type 2 diabetes mellitus holds an increased risk of RCC development [5, 6].

It is well known that mTOR inhibits cellular autophagy [33], another mechanism regulating cell viability and survival. Decreased cellular autophagy has been described in diabetic nephropathy with accumulation of peroxidated lipids in proximal tubules [4]. In rat diabetes related CCT, we found autophagy to be generally increased, as the surrogate marker LC3A [34] was detected in the cytoplasm. Of note, electron microscopy revealed membrane-bound autophagic vacuoles in the cytoplasm of CCT, whose volume fraction was augmented by NVP/BEZ235 treatment. Increased cellular autophagy has also been shown in human RCC cell lines [35] and is hypothesized as a counteracting effect of ccRCC cells to NVP/BEZ235 administration [36].

Glycogenotic lesions progress to clear or acidophilic cell RCC in the diabetes related and the NNM nephrocarcinogenesesis model of the rat at late stages after one to two years [7, 37], which is comparable to latency of tumorigenesis in the human situation, related to lifespan of species, whereas epidemiological data are missing.

Furthermore, our preliminary findings suggest that the distal tubular system (including the cortical part of the collecting ducts) is the most probable site of CCT, thus confirming previous results on renal clear cell lesions of rats [7, 13, 38] and humans [20]. CCT reveal in fact a cytoplasmic immunolabeling for CK7, an intermediate filament protein, which is typically expressed in human collecting ducts [39] and rat CCT lesions [7]. Additionally, CCT lack CD10 expression, similar to that described in human distal tubules and collecting ducts [40, 41]. Obviously, we are aware that these findings are not sufficient to claim a general designation of CCT to the distal tubular system. This interesting question was not the main issue of this study, but will be part of future investigations on human CCT.

In summary, the present data indicate that human glycogen storing CCT exhibit similar morphologic, metabolic, and molecular alterations to those that have been described in the rat model of diabetes related and chemically induced nephrocarcinogenesis. At the molecular level, the early activation of the PI3K/AKT/mTOR pathway was observed. Of note, the molecular and metabolic alterations as well as the proliferative activity driven by the PI3K/AKT/mTOR can be effectively restrained by the dual PI3K/mTOR inhibitor, NVP/BEZ235. Thus, activation of the PI3K/AKT/mTOR pathway in glycogenotic tubuli suggests a preneoplastic character of these lesions also in humans.

## MATERIALS AND METHODS

### Rat renal tissue

Housing of rats was described in detail previously [7, 21, 42] and was in accordance with the guidelines of the Society for Laboratory Animal Service and the German Animal Protection Law.

### Diabetes induced nephrocarcinogenesis

Rat kidney tissues and renal tumor specimens were obtained from previous short and long term experiments of 4 weeks and 24 months [7, 21, 42]. Briefly, diabetes was induced in adult inbred male Lewis rats (body weight 250–300 g) with a single subcutaneous dose of streptozotocin (80 mg/kg body weight) and was defined by a non-fasting blood-glucose level higher than 400 mg/dL. In short term experiments, groups of rats (*n* = 111) were subjected to daily oral administration of the PI3K/mTOR dual inhibitor, NVP/BEZ235 (kindly provided by Novartis, Basel, Switzerland), dissolved in 2% methylcellulose at a concentration of 40 mg/kg for one week or 10 mg/kg body weight for 4 weeks, respectively, or methylcellulose alone (control groups).

### N-Nitrosomorpholine (NNM) induced nephrocarcinogenesis

NNM was kindly provided by Prof. Dr. Rudolf Preussmann (German Cancer Research Center, Heidelberg, Germany). Rats received a single, high dose of NNM (orally, 200 mg NNM/kg body weight), as previously described [43], and sacrificed after 24 months.

### Tissue processing

Animals were killed under anaesthesia (100 mg/kg ketamine, 4 mg/kg xylazine) by perfusion fixation and tissue samples were processed for histology, immunohistochemistry (Supporting table 2), and electron microscopy as described in Supplementary Material and Methods.

### BrdU-labelling

Rats received four injections of 10 mg BrdU each, dissolved in 0.9% NaCl. Two injections were administered 24 and 1 h before sacrifice (s.c. and i.p.). The BrdU labelling index (BrdU-LI, i.e. the number of BrdU-labelled nuclei of 100 nuclei of the kidney tubular epithelium) of AEL, proximal (PT), and distal tubules (DT) was estimated at a magnification of ×400 by counting at least 2,000 nuclei per animal, respectively. PT were discriminated from DT due to their brush border and apical vacuoles at the luminal surface.

### Volume fraction determination of autophagic vacuoles (APV) in rat diabetes related CCT

At the ultrastructural level, autophagic vacuoles could be identified as membrane bound segregations of the cytoplasm. As they are filled with masses of glycogen and due to their very large size, we were able to detect them even at the light microscopy level. Semithin sections were cut and stained according to Richardson [44]. The volume fraction of APV was estimated from their area fraction at ×900 magnification, determined by the point-counting stereological technique with a monitor grid described by Weibel [45] using a NIKON DS-2MV digital camera and the NIKON NIS Elements Imaging Software Package 4.0. At least 500 points were counted within 3 to 5 visual fields per section, representing an area of 4.96 mm^2^. According to Weibel, these area fractions are equivalent to volume fractions.

### Human renal samples

Renal specimens were obtained from a total of 324 resected kidneys. Patients' informed consent was obtained in accordance with the approval of the local Institutional Review Board of the Universitätsmedizin Greifswald (Greifswald, Germany; BB 081/12a). A total of 102 cases of ccRCC were collected at the Institut für Pathologie, Universitätsspital Basel (Basel, Switzerland) and used for the construction of renal tissue microarrays (TMA). Tissue samples were processed for histology and immunohistochemistry (Supporting Table 2, 3) as described in Supplementary Material and Methods.

### Morphologic analysis

Glycogenotic tubules were identified as lesions of enlarged distal tubular epithelial cells with clear cytoplasm in H&E staining due to extensive glycogen storage, which stains deep purple in PAS staining [7, 8, 9]. The corresponding lesions in the enzyme- and immunohistochemical stained sections were detected by comparison with H&E-stained sections.

### Western blot analysis

ccRCC and corresponding non-tumor tissue were homogenized and processed as described in Supplementary Material and Methods. Nitrocellulose membranes were probed with specific primary antibodies (Supporting Table 1b).

### Cell lines

Treatment of human RCC cell lines Caki-1 and RCC4 with NVP-BEZ235 (mTOR/PI3K dual inhibitor, Novartis Pharmaceuticals, Basel, Switzerland) was performed as described in Supplementary Material and Methods, including proliferation and apoptosis assays and Western blot analysis with specific primary antibodies (Supporting Table 1a).

### Statistical analysis

Immunohistochemical expression of the proteins investigated in AEL and RCC was evaluated semiquantitatively by direct comparison with the adjacent parenchyma. BrdU-LI was compared intraindividually (AEL vs. DT and PT) and interindividually (NVPBEZ235 treated vs. untreated rats). Comparison of BrdU-LI and volume fraction of APV were tested using the paired or unpaired Wilcoxon-Mann-Whitney-U-test. Tuckey-Kramer test was used to assess the differences in proliferation and apoptosis among cell lines. Values of *p* < 0.05 were considered as statistically significant.

## SUPPLEMENTARY FIGURES AND TABLES


