# Supplementary material for: PI3K/AKT/mTOR pathway plays a major pathogenetic role in glycogen accumulation and tumor development in renal distal tubules of rats and men
Source: Oncotarget. 2015 Apr 16;6(15):13036–48. doi: 10.18632/oncotarget.3675 (PMC4536997; doi:10.18632/oncotarget.3675)
Supplement: Supplementary file 1 [file oncotarget-06-13036-s001.pdf]

SUPPLEMENTARY DATA

Processing of human and rat renal tissue

Samples of renal tumor and non-tumor tissue were rapidly processed after being explanted and fixed in buffered 4.5% formaldehyde, dehydrated, and embedded in paraffin. Serial sections of 2–3 µm thickness were stained with hematoxylin & eosin (H&E) and the periodic acid Schiff's reaction (PAS) for assessing the glycogen content. For immunohistochemistry and western blot analysis, slices of about 1.5 cm × 1.5 cm × 0.5 cm were immediately frozen in -120°C cold isopentane and stored at -80°C. Some specimens of 2 mm^3 were cut with a razor blade, fixed in 2.5% glutaraldehyde, embedded in Glycidether 100 (formerly called Epon 812), cut with diamond knifes with a Leica ultratome to 500 and 750 nm thick semi-thin slides and stained according to Richardson [22]. Ultrathin sections of 70–90 nm were stained with uranyl acetate and lead citrate and examined with a Libra 120 electron microscope from Carl Zeiss (Jena, Germany). Glycogenotic tubules were identified as lesions of enlarged distal tubule epithelial cells with pale cytoplasm in H&E staining due to extensive glycogen storage, which stains positive for PAS staining [1, 5, 6]. The corresponding lesions in the enzyme- and immunostained-sections were detected by comparison with H&E-stained sections.

Western blot analysis

Six ccRCC and corresponding non-tumor tissue were homogenized in lysis buffer containing the Complete Protease Inhibitor cocktail (Roche Molecular Biochemicals, Indianapolis, IN) and sonicated. Protein concentrations were measured with the Bio-Rad Protein Assay kit (Bio-Rad, Hercules, CA) using bovine serum as standard. For immunoblotting, aliquots of 40 µg were denatured by boiling in Tris-glycine SDS Sample buffer (Life Technologies, Grand Island, NY), separated by SDS-PAGE and transferred to nitrocellulose membranes by electroblotting. Membranes were blocked in 5% non-fat dry milk in Tris-buffered saline containing 0.1% Tween 20 for 1 h and probed with specific antibodies against AKT, p-AKT, Hexokinase 2, Pyruvate Kinase M2 (PKM2), Glyceraldehyde-3-phosphate-dehydrogenase (GAPDH), Lactate dehydrogenase (LADH A/C), fatty acid synthase (FASN), phosphorylated ATP citrate lyase (p-ACLY), acyl-CoA dehydrogenase for acyl chains of medium length (ACADM), squalene synthetase (SQS) (Supporting table 1b) followed by incubation with a secondary horseradish peroxidase-conjugated antibody.

Immunohistochemistry of rat CCT and tumors and human CCT (frozen sections)

Frozen tissue was cut in 10 µm thick slices and stained with H&E and PAS. Serial cryostat sections were also incubated with a battery of primary antibodies against proteins of the PI3K/AKT/mTOR signaling pathway and enzymes of glycolysis, de novo lipogenesis, and cholesterol synthesis (Supporting Table 2). Endogeneous peroxidase was quenched with 1% hydrogen peroxide, and positive reactivity of primary antibodies was identified using the Ultravision LP detection system HRP polymer and DAB as the chromogen substrate (Thermoscientific, Waltham, MA, USA). Immunohistochemical signal intensity in CCT and tumors was estimated semiquantitatevely comparing to corresponding surrounding unaltered renal tissue. Negative controls were stained without a primary antibody.

Immunohistochemistry of human CCT (paraffin sections)

Formalin-fixed, paraffin-embedded serial sections of five µm thickness were stained for cytokeratin 7 (mouse monoclonal anti-human cytokeratin 7 antibody, clone Ov-TL 12/30, dilution 1:100, DAKO, Hamburg, Germany), CD10 (monoclonal mouse anti-human CD10 antibody, dilution 1:25, DAKO, Hamburg, Germany) in an automated immunostainer (Leica Biosystems, Wetzlar, Germany) using a DAB (diaminobenzidine) kit, sections were counterstained with hematoxylin.

Tissue microarrays (TMAs) of human ccRCC

A total of 102 cases of ccRCC were collected at the Institut für Pathologie, Universitätsspital Basel (Basel, Switzerland) and used for the construction of renal TMAs. Tumor grade was defined according to the Fuhrman grading system: 4 cases were Fuhrman grade I; 28 cases Fuhrman grade II; 37 cases Fuhrman grade III and 32 cases Fuhrman grade IV. The study was approved by the Institutional Review Board of the Universities of Greifswald and Basel. Renal TMA was constructed using formalin-fixed, paraffin-embedded tissue. Paraffin blocks containing representative tumor areas were identified on corresponding H&E stained sections. The source block was cored, and a 1-mm core was transferred to the recipient "master block" using the Beecher Tissue Microarrayer (Beecher Instruments, Silver Spring, MD). 1–2 representative cores of tumor were arrayed per specimen.

Immunohistochemistry of TMAs

Immunohistochemical staining was performed for nine selected proteins belonging to the PI3K/AKT/mTOR pathway. Dilutions and manufacturers' information are listed in Supporting Table 3. Immunohistochemical staining was scored as positive if more than 10% of the tumor cells showed at least a focal weak cytoplasmatic and/or membranous staining. Staining patterns were compared to non-neoplastic renal cortical parenchyma.

Cell lines and treatments

The human RCC cell lines (Caki-1, Cell lines Service, Heidelberg, Germany; RCC4, Sigma-Aldrich, St. Louis, MO) were maintained in MEM (Caki-1; additional 1% Sodium Pyruvate 100 mM) and DMEM (RCC4; additional 1% L-Glutamine 200 mM and G418/Paneticin 5 µg/ml). Media contained 10% fetal bovine serum (FBS) and 1% Penicillin/Streptomycin. Cells were incubated 24 hours at 37°C at 5% CO2 in 96-well plates and grown for 48 hours. 10 or 20 µM, respectively, of NVP-BEZ235 (mTOR/PI3K dual inhibitor, Novartis Pharmaceuticals, Basel, Switzerland) was solubilized for *in vitro* assays in DMSO and was added to the medium and compared to DMSO incubated and untreated cells.

Proliferation and apoptosis assays

RCC cell lines were seeded at 2×10^3 cells/well in a 96-well plate, allowed to attach and adjust for the next 12 hours (corresponding to 0 hour time point in the graphs) and grown for additional 48 hours. Proliferation was assessed at these two time points with the BrdU Cell Proliferation Assay Kit (Cell Signaling Technology) by measuring the absorbance at 450 nm following the manufacturer's protocol. To measure apoptosis, cell lines were plated at 2×10^3 cells/well in a 96-well plate, incubated for 12 hours, subjected to 24 h serum deprivation (corresponding to 0 hour time point in the graphs), and continued to grow for additional 48 hours. Apoptosis was assessed using the Cell Death Detection ELISA-Plus Kit (Roche Molecular Biochemicals) by measuring the absorbance at 405 nm following manufacturer's instructions. Experiments were conducted at least three times in triplicate. Data are reported as percent viable or nonviable, respectively, tumor cells in each condition as compared with untreated cells or cells treated with DMSO alone.

Western blot analysis

Cells were pretreated as described above for ccRCC. After transfer of isolated proteins membranes were probed with specific antibodies against AKT, p-AKT, phosphorylated eukaryotic translation initiation factor 4E binding protein 1 (p-4E-BP1), phosphorylated ribosomal protein S6 (p-RPS6) and fatty acid synthase (FASN) (Supporting table 1a) followed by incubation with a secondary horseradish peroxidase-conjugated antibody.

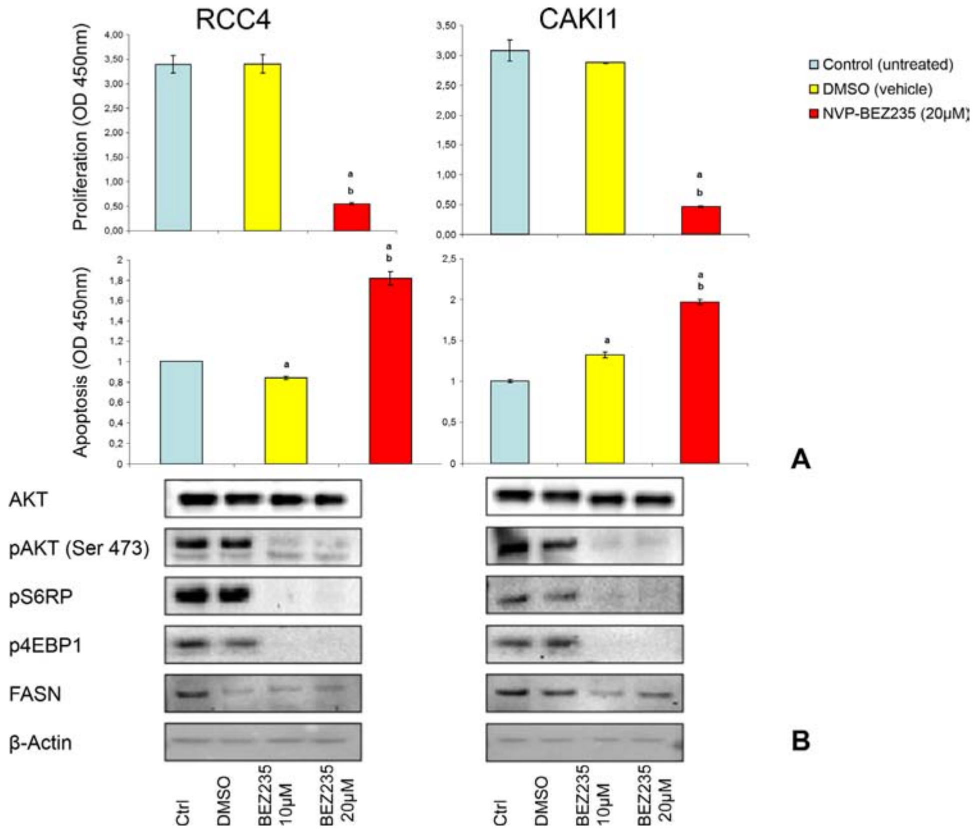

Supplementary Figure S1: Effects of NVP/BEZ235 on proliferation, apoptosis and the AKT/mTOR pathway in RCC4 and CAKI1 cell lines. A. Incubation of RCC4 and CAKI1 cell lines with the dual PI3K/mTOR inhibitor NVP/BEZ235 (20 µM, 48 hours) clearly reduces cell proliferation and increases apoptosis in comparison to untreated (Control) and vehicle treated (DMSO) cells. Proliferation and apoptosis were assessed using the BrdU Cell Proliferation Assay and Cell Death Assay ELISAplus Kit, respectively, following the manufacturer`s instructions. Data represent means ± standard deviation; a, vs. control, and b, vs. DMSO; $p < 0.01$. **B.** Treatment of RCC4 and CAKI1 cell lines with NVP/BEZ235 (10 or 20 µM) diminishes intracellular activated/phosphorylated AKT and its downstream targets, as assessed by Western blot analysis.

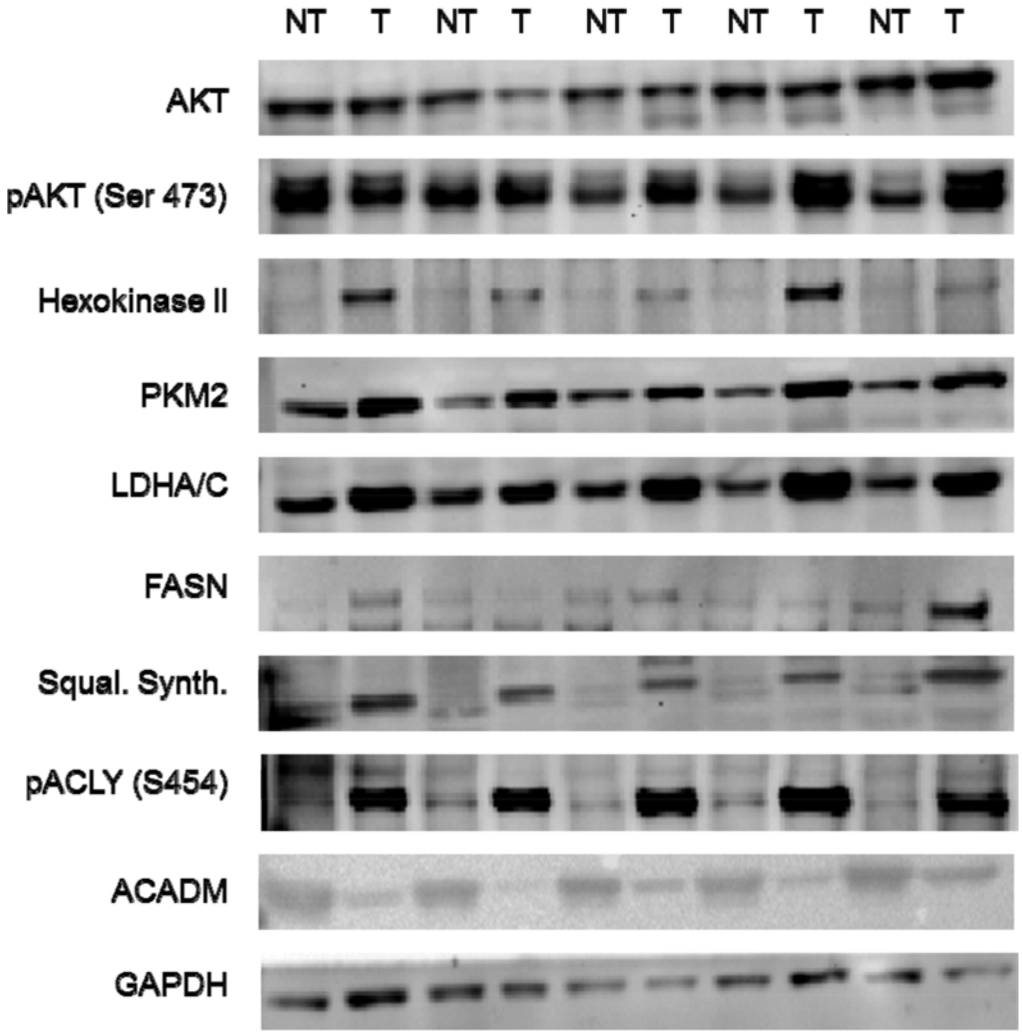

Supplementary Figure S2: Metabolic events in human renal clear cell carcinoma and corresponding unaltered renal non-tumorous tissue. Activation of glycolysis [Hexokinase 2, Pyruvate Kinase M2 (PKM2), Glyceraldehyde-3-phosphate-dehydrogenase (GAPDH), Lactate dehydrogenase (LADH A/C)], fatty acid synthesis [fatty acid synthase (FASN) and activated/phosphorylated ATP citrate lyase (pACLY)], and cholesterol synthesis (squalene synthetase (SQS)] and reduction of lipid β-oxidation [(acyl-CoA dehydrogenase for acyl chains of medium length (ACADM)] in clear cell carcinoma samples in comparison to related non tumor tissue. Whole cell lysates were prepared from tumor (T) and non-tumorous (NT) tissue and immunoblotted with indicated antibodies.

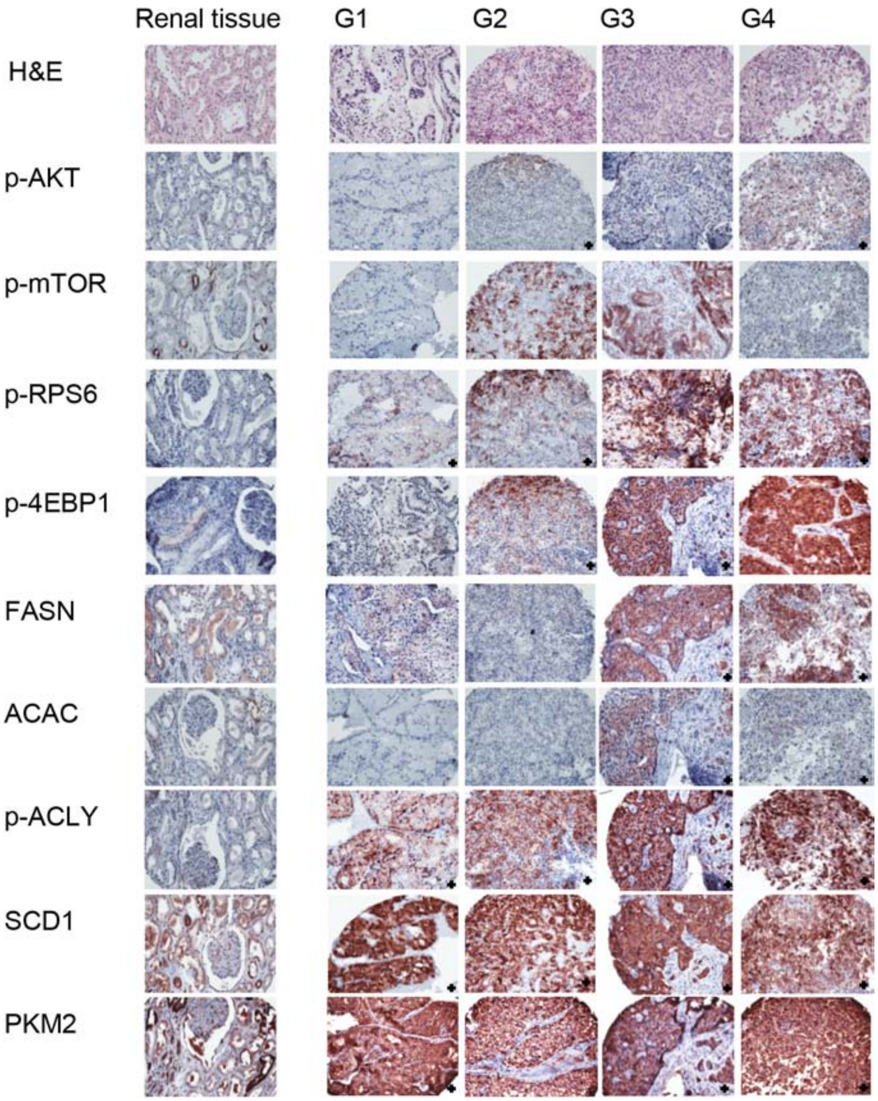

Supplementary Figure S3: Activation of the PI3K/AKT/mTOR pathway and induction of related lipogenesis and glycolysis in tissue microarrays of human clear cell renal cell carcinoma. Representative immunohistochemical staining of Fuhrmann grades 1 to 4, compared to non-neoplastic renal tissue (left column): ACAC, acetyl-Coenzyme A carboxylase; ACLY, ATP citrate lyase; AKT, v-akt murine thymoma viral oncogene homolog; 4E-BP1, eukaryotic translation initiation factor 4E binding protein 1; FASN, fatty acid synthase; mTOR, mammalian target of Rapamycin; PKM2, pyruvate kinase M2; RPS6, ribosomal protein S6; SCD1, stearoyl-CoA desaturase. Positive scored staining (more than 10% of the tumor cells showed at least a focal weak cytoplasmatic and/or membranous staining) are tagged +.

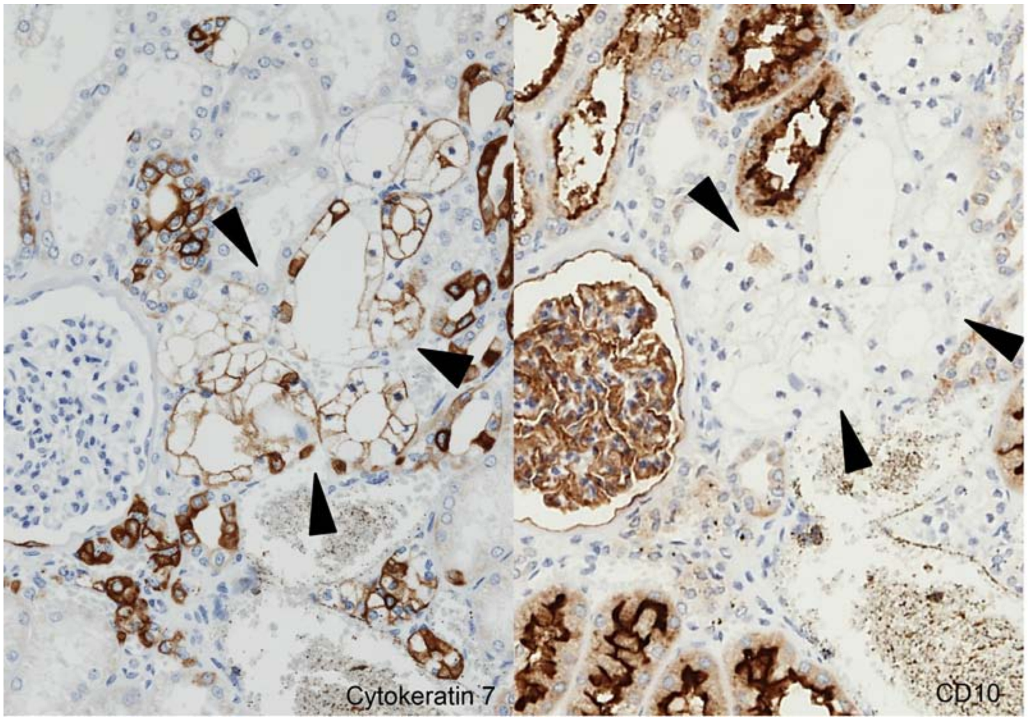

Supplementary Figure S4: Expression pattern of cytokeratin 7 and CD10 in human clear cell tubules (CCT). Enlarged cells of CCT (arrowheads) exhibit CK7 immunoreactivity in the periphery of their glycogenotic cytoplasm, just like neighboring not altered distal tubular epithelium. CD10 is not detectable in CCT, but in neighboring proximal tubulus epithelium. Length of the lower edge 0.375 mm.

Supplementary Table S1a. List of primary antibodies used for immunoblotting of human ccRCC cell lines

Protein	dilution	antibody
AKT	1:300	rabbit monoclonal[1]
FASN	1:1000	mouse monoclonal[2]
p-AKT	1:300	rabbit monoclonal[1]
p-RPS6	1:300	rabbit monoclonal[1]
p-4EBP1	1:300	rabbit monoclonal[1]

[1] Cell Signaling Technology Inc (Danvers, MA)
[2] BD Biosciences (San Diego, CA)

Supplementary Table S1b. List of primary antibodies used for Western blot analysis of human ccRCC samples

Protein	dilution	antibody
ACADM	1:300	rabbit monoclonal[1]
AKT	1:300	rabbit polyclonal[2]
FASN	1:1000	mouse monoclonal[3]
GAPDH	1:100	rabbit polyclonal[4]
Hexokinase 2	1:300	rabbit monoclonal[2]
LADHA/C	1:300	rabbit monoclonal[2]
p-ACLY	1:300	rabbit polyclonal[2]
PKM2	1:300	rabbit monoclonal[2]
SQS	1:100	rabbit monoclonal[4]

[1] Abcam (Cambridge, UK)
[2] Cell Signaling technology Inc (Danvers, MA)
[3] BD Biosciences (San Diego, CA)
[4] Santa Cruz Biotechnology (Santa Cruz, CA)

Supplementary Table S2. List of primary antibodies used for immunohistochemistry in cryostat sections

Protein	dilution	antibody
ACAC	1/100	rabbit monoclonal[4]
ACADM	1/100	rabbit monoclonal[1]
AMPK α1 α2	1/100	rabbit polyclonal[1]
ChREBP	1/25	rabbit polyclonal[5]
ERK1/2	1/100	mouse monoclonal[2]
FAS	1/100	mouse monoclonal[2]
GLUT1	1/100	rabbit polyclonal[1]
GLUT2	1/100 ON	rabbit polyclonal[6]
GLUT4	1/25	mouse monoclonal[1]
IGLK	1/25	rabbit polyclonal[1]
Insulin receptor	1/25	rabbit polyclonal[6]
IRS1	1/25	rabbit polyclonal[6]
LC3A	1/6400	rabbit monoclonal[4]
MEK-1	1/25	rabbit polyclonal[6]
MKP-3	1/25	rabbit polyclonal[6]
MVK	1/100 ON	rabbit[7]
p-ACLY	1/100	rabbit polyclonal[4]
Pan-AKT	1/100 ON	rabbit monoclonal[4]
p-GSK-3β	1/100 ON	rabbit monoclonal[4]
PFKL	1/25	rabbit polyclonal[6]
PKM2	1/100 ON	rabbit monoclonal[4]
p-mTOR	1/100 ON	rabbit monoclonal[4]
p-RPS6	1/100	rabbit polyclonal[4]
p-4EBP1	1/100	rabbit monoclonal[4]
Ral A	1/100 ON	mouse monoclonal[2]
SCD-1	1/100	rabbit polyclonal[6]
SREBP-1	1/100 ON	rabbit polyclonal[6]
USP-2	1/25 ON	rabbit[7]

[1] abcam (Cambridge, UK)
[2] BD Biosciences (San Diego, CA)
[3] BioLegend, (San Diego, CA)
[7] Sigma-Aldrich (Germany)
[4] Cell Signaling technology Inc (Danvers, MA)
[5] Novus Biologicals (Littleton, CO)
[6] Santa Cruz Biotechnology (Santa Cruz, CA)
[8] RnD Systems (Minneapolis, MN)
ON: over night incubation
[*] For detecting the primary goat antibody with the Ultravision LP detection system (Thermoscientific, Waltham, MA, USA), a bridging rabbit-anti-goat antibody (KPL, Gaitherburg, USA) was used.

Supplementary Table S3. List of primary antibodies used for immunohistochemistry in paraffin embedded tissue microarrays

Protein	Dilution	Antibody
ACAC	1:100	rabbit monoclonal[1]
FASN	1:1000	mouse monoclonal[2]
p-ACLY	1:100	rabbit polyclonal[1]
Pan-AKT	1:100	rabbit monoclonal[1]
PKM2	1:100	rabbit monoclonal[1]
p-mTOR	1:50	rabbit monoclonal[1]
p-RPS6	1:300	rabbit polyclonal[1]
p-4EBP1	1:500	rabbit monoclonal[1]
SCD1	1 :100	rabbit polyclonal[1]

[1] Cell Signaling Technology Inc (Danvers, MA)
[2] BD Biosciences (San Diego, CA)

Supplementary Table S4. Total number and percentage of evaluable ccRCCs (tumor grades G1-G4) with membraneous or/ and cytoplasmatic staining using antibodies against respective proteins

	pAKT	pmTOR	pERK	p4EBP1	pRPS6	FASN	ACAC	ACLY	SCD	PKM2
G1<br>*N* = 4	0/4<br>0.0%	2/4<br>50.0%	3/4<br>75.0%	2/4<br>50.0%	4/4<br>100.0%	2/4<br>50.0%	2/4<br>50.0%	4/4<br>100.0%	4/4<br>100.0%	4/4<br>100.0%
G2<br>*N* = 28	8/26<br>30.8%	17/24<br>70.8%	20/27<br>74.1%	15/17<br>88.2%	17/24<br>70.8%	12/21<br>57.1%	6/22<br>27.3%	19/20<br>95%	27/27<br>100.0%	21/21<br>100.0%
G3<br>*N* = 37	11/34<br>32.4%	23/35<br>65.7%	33/35<br>94.3%	25/26<br>96.1%	30/33<br>90.9%	22/33<br>66.7%	12/32<br>37.5%	29/32<br>90.6%	35/36<br>100.0%	33/33<br>100.0%
G4<br>*N* = 32	17/31<br>54.9%	18/29<br>62.1%	26/30<br>86.7%	19/20<br>95%	26/27<br>96.3%	20/25<br>80.0%	8/22<br>36.4%	23/25<br>92.0%	28/28<br>100.0%	23/23<br>100.0%
